# High Density Lipoproteins Inhibit Oxidative Stress-Induced Prostate Cancer Cell Proliferation

**DOI:** 10.1038/s41598-018-19568-8

**Published:** 2018-02-02

**Authors:** Massimiliano Ruscica, Margherita Botta, Nicola Ferri, Eleonora Giorgio, Chiara Macchi, Guido Franceschini, Paolo Magni, Laura Calabresi, Monica Gomaraschi

**Affiliations:** 1 Dipartimento di Scienze Farmacologiche e Biomolecolari, Università degli Studi di Milano, Milano, Italy; 2 Dipartimento di Scienze del Farmaco, Università degli Studi di Padova, Padova, Italy; 3 Centro Enrica Grossi Paoletti, Dipartimento di Scienze Farmacologiche e Biomolecolari, Università degli Studi di Milano, Milano, Italy

## Abstract

Recent evidence suggests that oxidative stress can play a role in the pathogenesis and the progression of prostate cancer (PCa). Reactive oxygen species (ROS) generation is higher in PCa cells compared to normal prostate epithelial cells and this increase is proportional to the aggressiveness of the phenotype. Since high density lipoproteins (HDL) are known to exert antioxidant activities, their ability to reduce ROS levels and the consequent impact on cell proliferation was tested in normal and PCa cell lines. HDL significantly reduced basal and H_2_O_2_-induced oxidative stress in normal, androgen receptor (AR)-positive and AR-null PCa cell lines. AR, scavenger receptor BI and ATP binding cassette G1 transporter were not involved. In addition, HDL completely blunted H_2_O_2_-induced increase of cell proliferation, through their capacity to prevent the H_2_O_2_-induced shift of cell cycle distribution from G0/G1 towards G2/M phase. Synthetic HDL, made of the two main components of plasma-derived HDL (apoA-I and phosphatidylcholine) and which are under clinical development as anti-atherosclerotic agents, retained the ability of HDL to inhibit ROS production in PCa cells. Collectively, HDL antioxidant activity limits cell proliferation induced by ROS in AR-positive and AR-null PCa cell lines, thus supporting a possible role of HDL against PCa progression.

## Introduction

In almost all Western countries, prostate cancer (PCa) is the most commonly diagnosed cancer and the second leading cause of cancer-related death in men^1^. Since the prostate is an androgen-dependent organ, PCa development is tightly associated with the presence of androgens and the activation of the androgen receptor (AR)^2^. Thus, AR is considered the most relevant target to control the growth and dissemination of PCa, with androgen deprivation (ADT) representing the backbone of the therapy for locally advanced and metastatic PCa after failure of localized treatments^3^. However, after initial effective response to ADT, PCa may develop into a castration-resistant phenotype (CRPC) despite low levels of circulating androgens^4^. In some cases, CRPC bypasses the requirements for AR signalling, while in others it retains its dependence on AR signalling as primary oncogenic driver^5^. To date, CRPC has few therapeutic options resulting only in a limited survival prolongation. Thus, novel strategies that could have direct cytotoxic effects on tumour cells or that could modify cell biology, making tumour cells more sensitive to the action of classical cytotoxic agents are required.

Recent evidence suggests that oxidative stress can play a role in the pathogenesis and the progression of PCa^6^. Oxidative stress occurs when the balance between the production of pro-oxidant molecules, as reactive oxygen species (ROS), and their neutralization by detoxifying systems is lost. ROS are a heterogeneous group of highly reactive ions and molecules derived from molecular oxygen, including superoxide anion, hydroxyl radicals, hydrogen peroxide and singlet oxygen^7^. ROS are normally generated within cell mitochondria, peroxisomes and microsomes; indeed, they are a by-product of normal mitochondrial respiration and of other enzymes as NADPH oxidase, xanthine oxidase and lipoxygenases^7^. Interestingly, ROS generation is higher in PCa cells than in normal prostate epithelial cells and this increment is proportional to the aggressiveness of the phenotype^8^. In addition, exogenous sources of ROS can be present in tumour microenvironment as xenobiotics or infiltrating inflammatory cells^9^. Indeed, resident immune cells, as lymphocytes, mast cells and macrophages, or those infiltrating during an inflammatory event, utilize ROS and pro-oxidant enzymes to attack and neutralize a foreign intruder^10^. PCa promotion and progression by oxidative stress are likely due to ROS reactivity towards key cellular components as nucleic acids, proteins and lipids. ROS can directly attack DNA causing single or double strand breaks as well as pyrimidine and purine lesions, both of which can affect the integrity of the genome and genomic instability^11^. In addition, ROS may cause epigenetic alterations, as DNA methylation patterns, possibly leading to the activation of oncogenes and/or the inhibition of tumour-suppressor genes^11^. ROS can also affect several signalling pathways mediating cell proliferation and differentiation, invasion and angiogenesis; for example, ROS were shown to activate the MAPK and PI3K/Akt pathways, to promote the production of prostaglandin E2 and of matrix metalloproteinases^12,13^.

High density lipoproteins (HDL) are a heterogeneous family of lipoproteins whose anti-atherosclerotic properties are well recognized^14^. Atheroprotection by HDL is related to their capacity to promote the removal of cholesterol from peripheral cells and its transport to the liver for excretion through the bile among the so-called reverse cholesterol transport^15^. In addition, HDL display anti-inflammatory and antioxidant activities that can contribute to their atheroprotective effects^16^. Many HDL activities are mediated by their interaction with different transmembrane proteins, as the transporters ATP-binding cassette A1 and G1 and the scavenger receptor type BI^15,16^. Antioxidant properties of HDL are mainly due to: (i) their ability to uptake oxidized lipids from cell membranes and other lipoproteins, (ii) the presence on HDL of antioxidant enzymes as paraoxonase (PON-1), lecithin:cholesterol acyltransferase (LCAT) and platelet-activating factor acetylhydrolase, and (iii) their main protein components, apolipoproteins A-I and A-II, which can participate to redox reactions undergoing oxidation on their methionine residues^14^. HDL purified from human plasma are unsuitable for drug development, because of safety concerns, problems related to large-scale production and HDL heterogeneity^17^. These limitations can be overcome by preparing synthetic HDL (sHDL), discoidal particles made with a purified apolipoprotein and synthetic phospholipids; sHDL have been shown to retain several atheroprotective activities of plasma-derived HDL *in vitro* and *in vivo* and are currently under development as anti-atherosclerotic agents^18^.

Few and inconclusive studies addressed the relationship between plasma levels of HDL-C and the risk or prognosis of PCa, while contrasting data have been published on the effect of HDL on the proliferation of PCa cell lines^19^. In the present study, we investigated the ability of HDL to reduce oxidative stress in PCa cells and the consequent impact on cell proliferation. To address tumour heterogeneity composed of cells with various degree of transformation, experiments were performed on normal prostate epithelial cells, AR-positive and AR-null PCa cell lines.

## Results

### Characterization of isolated HDL

Proteins accounted for about 43% of total HDL mass; phospholipids and cholesteryl esters represented most of the HDL lipids, with minor amounts of unesterified cholesterol and triglycerides (Table S1)^20^. HDL protein fraction was composed by 78% of apoA-I and by 18% of apoA-II (Table S1). Regarding antioxidant enzymes carried by HDL, LCAT concentration was 35.2 ± 3.1 μg/mg of HDL protein, but no LCAT activity could be detected in isolated HDL. PON-1 activity in isolated HDL was 16.3 ± 3.6 nmol/min/mg of protein; this value is less than 5% of the normal activity, as assessed in our laboratory on plasma samples from control subjects (352.3 ± 24.5 nmol/min/ml).

### HDL reduced basal and H_2_O_2_-induced oxidative stress in LNCaP and PC-3 cells

Consistent with previous findings^8^, basal oxidative stress was significantly higher in PCa cell lines compared to normal prostate epithelial cells and the observed increase was proportional to the aggressiveness of the phenotype. Indeed, basal ROS level was 1.4 fold higher in LNCaP and 2.6 fold higher in PC-3 compared to that observed in PNT2 cells (Fig. S1).

**Figure 1:**
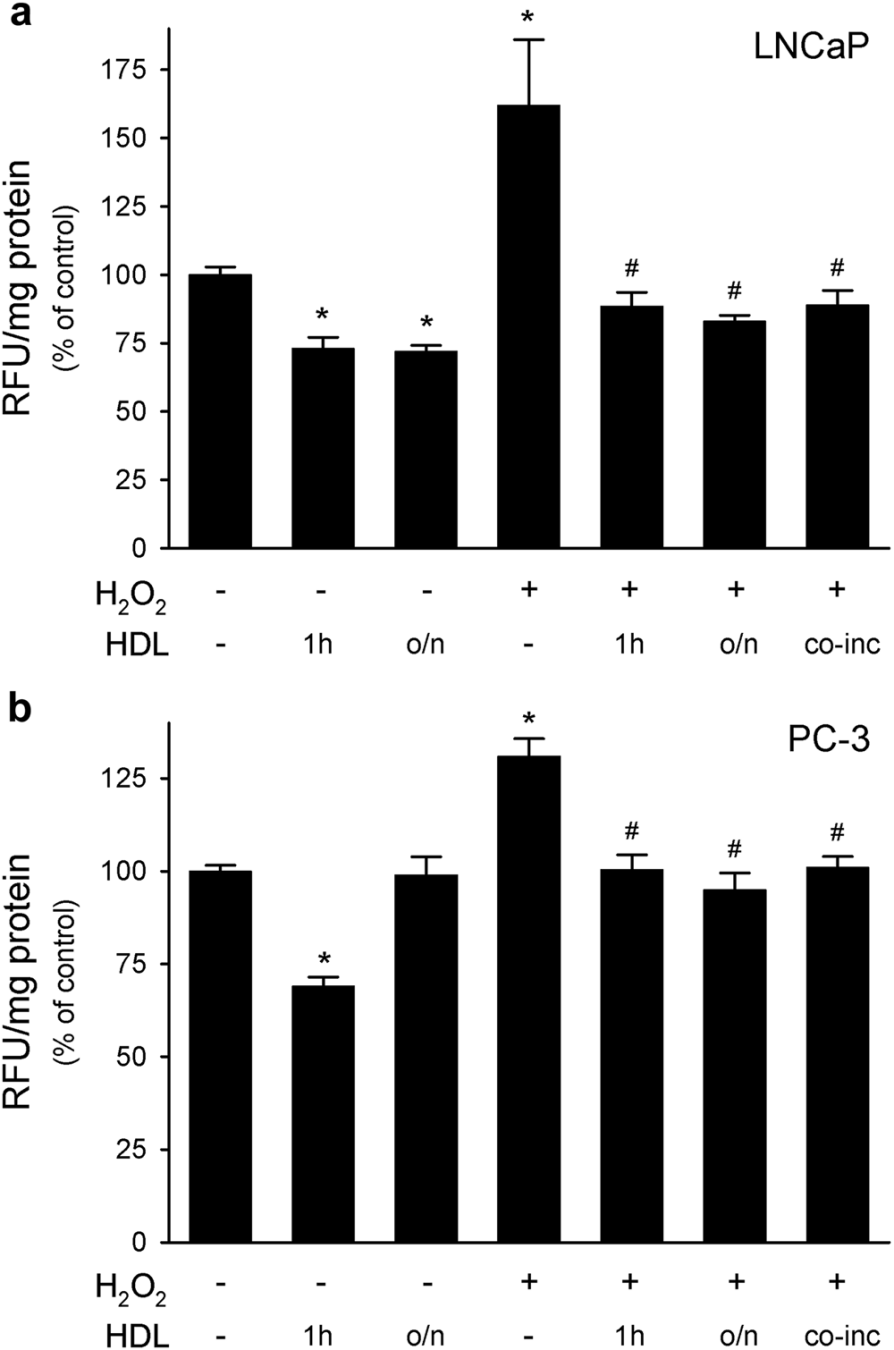
HDL reduced ROS production in LNCaP and PC-3 cell lines. LNCaP (Panel a) and PC-3 cells (Panel b) were pre-treated overnight (o/n) or for 1 h with HDL 0.5 mg/ml and then stimulated or not with 0.5 mM H_2_O_2_ for 1 h. The effect of the co-incubation of HDL with H_2_O_2_ (co-inc) was also tested. ROS production was evaluated by fluorescence. Data are expressed as relative fluorescent units (RFU) normalized by the protein concentration of total cell lysate, mean ± SD, n = 4. *P < 0.05 vs control, ^#^*P* < 0.05 vs H_2_O_2_-stimulated cells.

Short- or long-term exposure to HDL reduced basal oxidative stress in LNCaP. Indeed, the incubation of LNCaP with HDL significantly decreased basal ROS level when given overnight (−28%) or only for 1 h before ROS detection (−27%, Fig. 1a). HDL were also able to reduce basal oxidative stress in PC-3 cells when given 1 h before ROS detection (−31%), but not for a longer incubation, likely as a consequence of the higher ROS production in this cell type (Fig. 1b).

The ability of HDL to inhibit ROS production was also tested in cells stimulated with H_2_O_2_ for 1 h, in order to mimic a pro-oxidant environment. In LNCaP, H_2_O_2_ induced a 1.6-fold increase in basal ROS production, but this rise was completely blunted when cells were co-incubated with HDL (Fig. 1a). Interestingly, when HDL were added overnight or only for 1 h and then removed before H_2_O_2_ stimulation, ROS increase was again completely blunted. The anti-oxidant effect of HDL was concentration-dependent and 0.5 mg of protein/ml was the lowest concentration that significantly reduced ROS levels in H_2_O_2_-stimulated cells (Fig. S2). HDL were also able to exert their antioxidant effects on PC-3 cells: the 1.3-fold increment in ROS production induced by H_2_O_2_ was prevented by all treatments with HDL (Fig. 1b). Similar results were obtained with the normal prostate epithelial cell line PNT2 (Fig. S4).

### AR activation was not involved in HDL antioxidant effect

**Figure 2:**
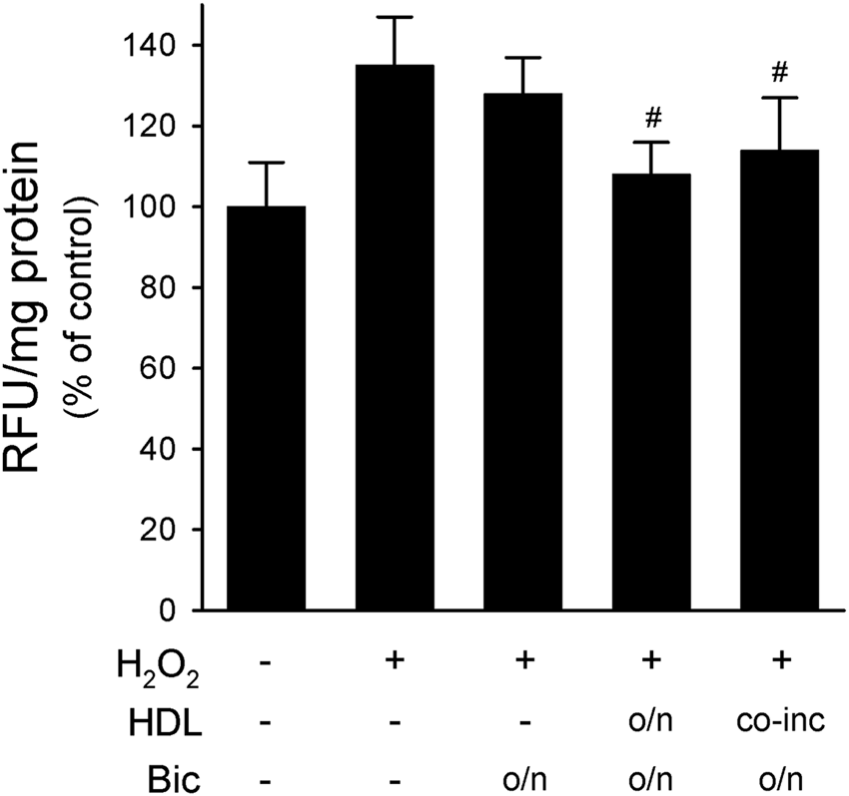
AR activation is not involved in HDL antioxidant effect. LNCaP were treated overnight (o/n) with the androgen receptor antagonist bicalutamide (100 nM) before stimulation with 0.5 mM H_2_O_2_ for 1 h. HDL at 0.5 mg/ml were given overnight or as co-incubation with H_2_O_2_ (co-inc) for 1 h. ROS production was evaluated by fluorescence. Data are expressed as relative fluorescent units (RFU) normalized by the protein concentration of total cell lysate, mean ± SD, n = 6. ^#^*P* < 0.05 vs H_2_O_2_-stimulated cells.

The AR antagonist bicalutamide was used to disclose the role of AR signalling in mediating ROS production and HDL antioxidant effect in LNCaP. Overnight incubation with bicalutamide did not modify ROS increase after H_2_O_2_ challenge; indeed, ROS levels in cells pre-treated with bicalutamide and then stimulated with H_2_O_2_ for 1 h were comparable to those of untreated H_2_O_2_-stimulated cells (Fig. 2). HDL retained their antioxidant effect on H_2_O_2_-stimulated cells even in the presence of bicalutamide. Indeed, when LNCaP were pre-treated with bicalutamide and then stimulated with H_2_O_2_, HDL were able to significantly reduce ROS levels as an o/n pre-treatment or as a co-incubation with H_2_O_2_ (Fig. 2).

### HDL antioxidant effect was not mediated by ABCG1 and SR-BI or by the modulation of cell cholesterol content

**Figure 3:**
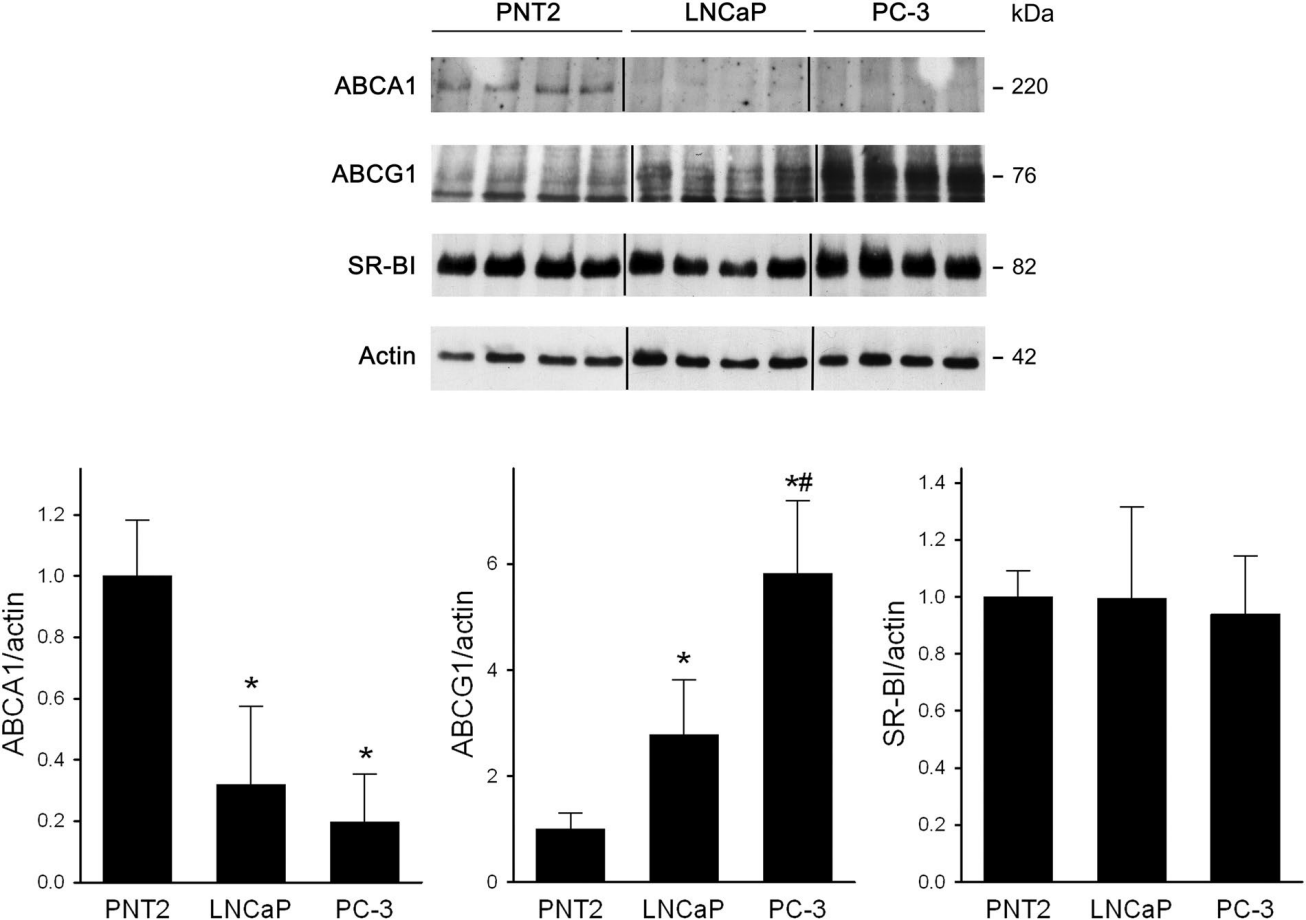
Expression of ABCA1, ABCG1 and SR-BI in prostate cell lines. Protein expression was analyzed by western blotting in PNT2, LNCaP and PC-3 cell lines. Results of the densitometric analysis are expressed as fold increase of the target/β-actin ratio in tumour cell lines vs PNT2 cells, mean ± SD, n = 7. Representative blots are shown at the top. Images have been manipulated to change the original loading sequence LNCaP-PNT2-PC3 into the more appropriate PNT2-LNCaP-PC3. Images are from the same blots and same exposures. *P < 0.05 vs PNT2, ^#^*P* < 0.05 vs LNCaP.

First, LNCaP and PC-3 cells were tested to evaluate altered protein expression of the transporters ABCA1 and ABCG1, and of the receptor SR-BI when compared to the normal prostate epithelial cell line PNT2 (Fig. 3). ABCA1 showed a very low expression in PNT2 cells and a further decrease was evident in both PCa cell lines (−68% in LNCaP and −81% in PC-3). ABCG1 was almost undetectable in PNT2 cells, but a significant increase was observed in PCa cell lines; in particular, a 2.8 fold increase in LNCaP and a 5.8 fold increase in PC-3 were detected. SR-BI was well expressed in PNT2 cells and no significant changes were observed in the two PCa cell lines.

**Figure 4:**
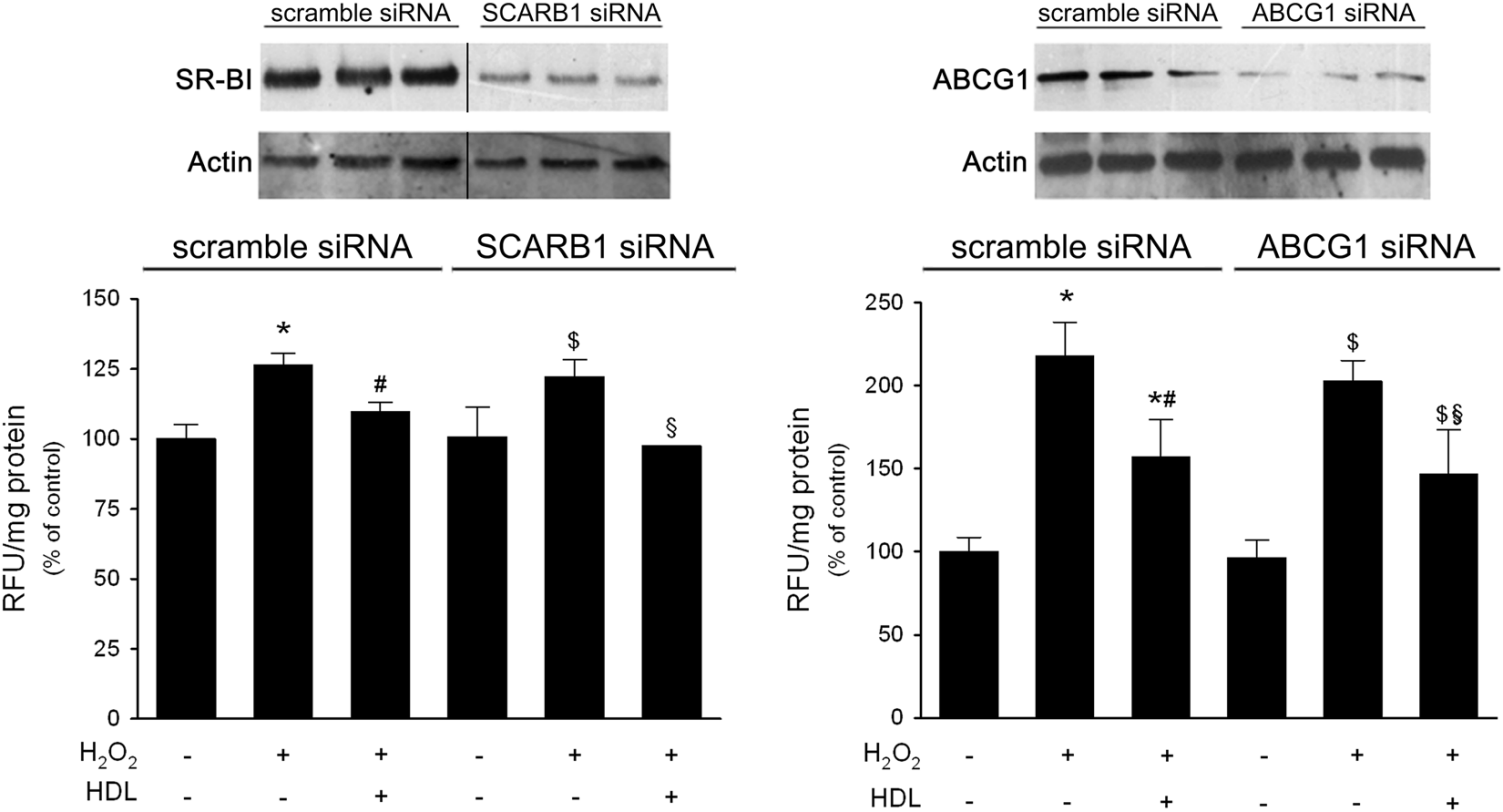
SR-BI and ABCG1 are not involved in HDL antioxidant effect. Panel A, SR-BI and ABCG1 protein levels in PC-3 cells treated with specific or control (scrambled) siRNA. Image of SR-BI blot has been manipulated to eliminate empty lanes between control and SCARB1 siRNA samples and to show 3 bands/group as the original non manipulated blot for ABCG1. Images come from the same blots and same exposures. Original images of SR-BI are reported in Fig. S5. Panel B, ROS levels in H_2_O_2_-stimulated PC-3 cells pre-treated with specific or control (scrambled) siRNA. The effect of the co-incubation of 0.5 mM H_2_O_2_ with 0.5 mg/ml HDL for 1 h was tested. ROS production was evaluated by fluorescence. Data are expressed as relative fluorescent units (RFU) normalized by the protein concentration of total cell lysate, mean ± SD, n = 3. *P < 0.05 vs control cells incubated with scrambled siRNA, ^#^*P* < 0.05 vs H_2_O_2_-stimulated cells incubated with scrambled siRNA, ^$^*P* < 0.05 vs control cells incubated with ABCG1 or SCARB1 siRNA, ^§^*P* < 0.05 vs H_2_O_2_-stimulated cells incubated with ABCG1 or SCARB1 siRNA.

The possible role of the SR-BI receptor and of the ABCG1 transporter in mediating HDL antioxidant effect was disclosed through RNA interference experiments in PC-3 cells. The treatment with specific siRNAs caused a marked inhibition of SR-BI and ABCG1 protein expression; indeed, SR-BI protein expression was reduced by 77.0 ± 8.9% and ABCG1 by 76.0 ± 8.3% (Fig. 4). The severe reduction of SR-BI expression affected neither the extent of ROS levels after H_2_O_2_ treatment, nor the antioxidant effect of HDL. Similar results were obtained when ABCG1 was silenced (Fig. 4). Being ABCA1 almost undetectable in tumour cells, silencing experiments targeting this transporter were not performed.

To further exclude that the antioxidant effect of HDL was mediated by the modulation of cell cholesterol content, experiments were repeated after cholesterol loading by LDL or depletion by βMCD. LDL increased cell cholesterol by 43.7 ± 7.6% in LNCaP and by 52.8 ± 22.7% in PC-3, while βMCD caused a reduction of 19.9 ± 1.4% in LNCaP and of 22.2 ± 8.8% in PC-3. However, these changes of cell cholesterol did not affect basal or H_2_O_2_-stimulated ROS levels in both cell lines; in addition, HDL antioxidant effect was not altered by previous treatment with LDL or βMCD (Fig. S3).

### HDL inhibited H_2_O_2_-induced cell proliferation

**Figure 5:**
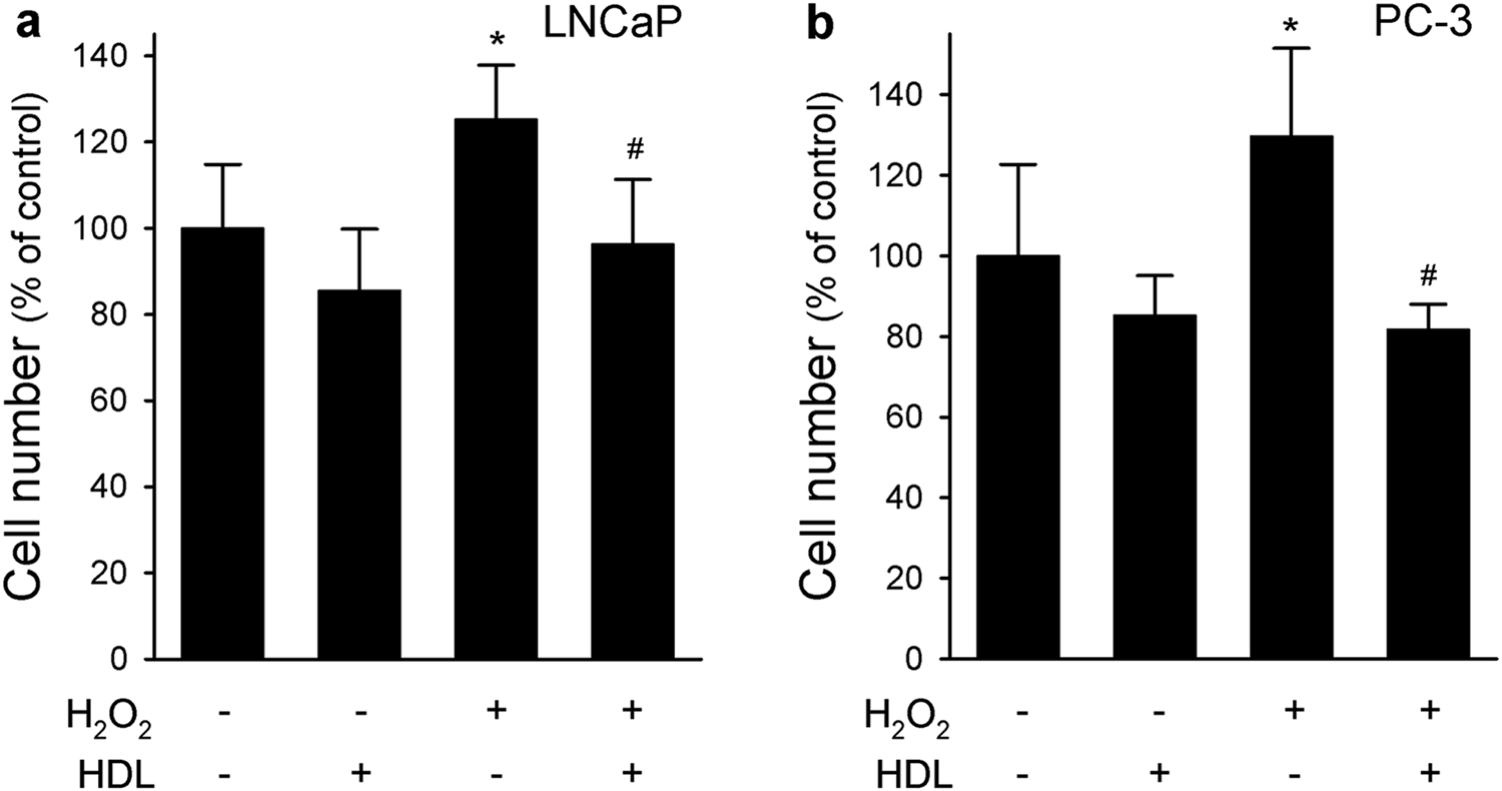
HDL inhibit cell proliferation induced by H_2_O_2_ in LNCaP and PC-3 cell lines. LNCaP (Panel a) and PC-3 cells (Panel b) were incubated for 72 h with H_2_O_2_ 5μM, HDL 0.5 mg/ml or with a combination of both. Cells were then harvested and counted in a Burker chamber. Data are expressed as mean ± SD, n = 4. *P < 0.05 vs control, ^#^*P* < 0.05 vs H_2_O_2_-stimulated cells.

To mimic a chronic low-grade pro-oxidant environment, LNCaP were exposed to 5 µM H_2_O_2_ for 72 h; this treatment resulted in a significant increase of cell count, which raised by 25.3% (Fig. 5a). The co-incubation with HDL significantly reduced H_2_O_2_-mediated rise of cell count by 23.1%, bringing cell proliferation back to values of untreated cells (Fig. 5a). Similarly, H_2_O_2_ stimulated PC-3 cell growth by 29.7%, an effect completely blunted by the co-incubation with HDL (−36.9% vs H_2_O_2_) (Fig. 5b). Interestingly, the treatment with HDL alone tended to decrease basal cell proliferation in both PCa lines, although the effect was far from statistical significance (LnCaP −14.4% vs untreated cells; PC-3 −14.7% vs untreated cells) (Fig. 5). Data were confirmed by MTT assay in both cell lines (not shown).

### HDL blunted H_2_O_2_-induced shift of cell cycle towards G2/M phase

**Figure 6:**
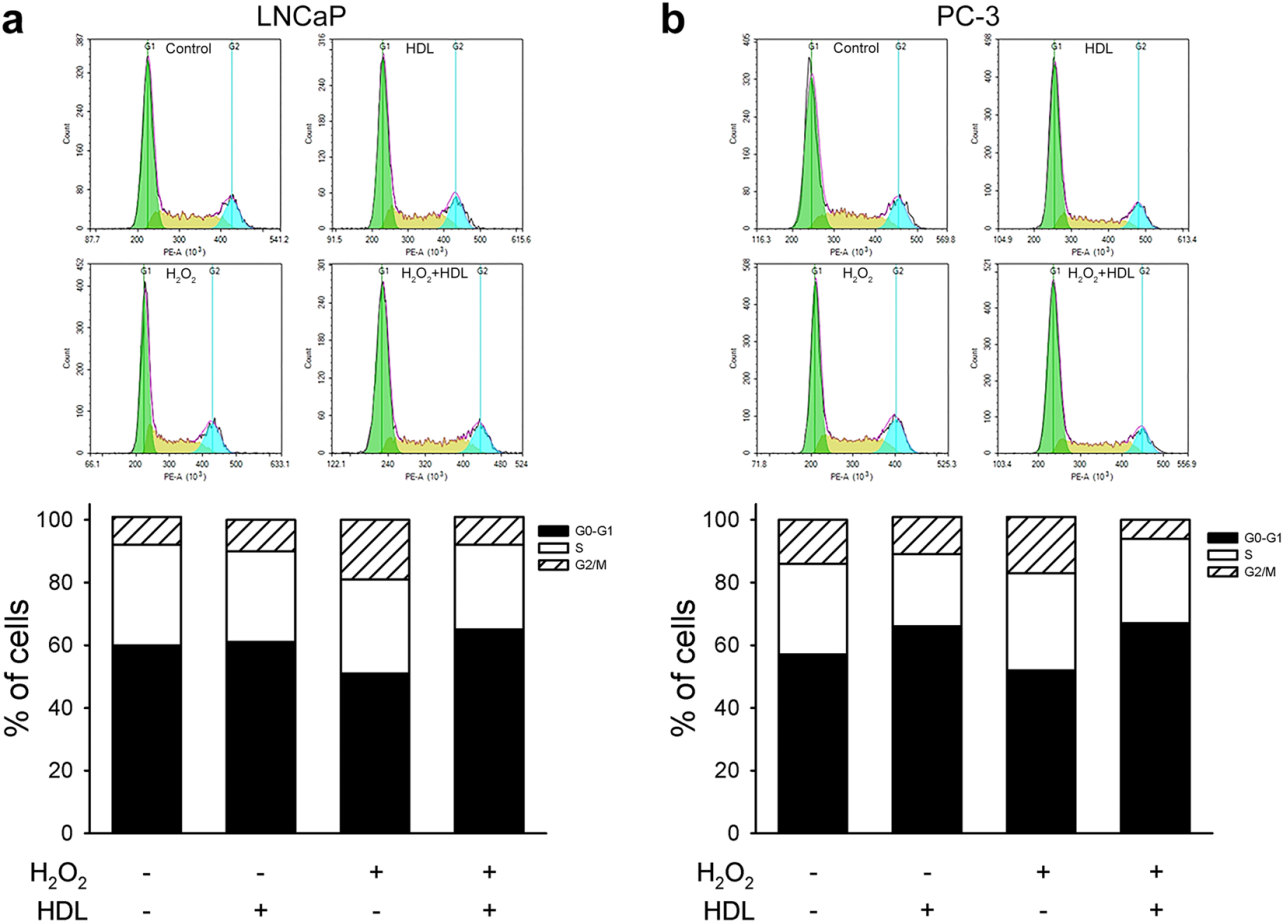
HDL modulate cell cycle distribution in H_2_O_2_-treated cells. LNCaP (Panel a) and PC-3 cells (Panel b) were incubated for 72 h with H_2_O_2_ 5μM, HDL 0.5 mg/ml or with a combination of both. Cells were then harvested, stained with propidium iodide and subjected to FACS analysis. Representative histograms of each experimental group and cumulative results of the percentage distribution along cell cycle (n = 3) are shown.

If compared to untreated cells, the exposure of LNCaP to H_2_O_2_ for 72 h caused an alteration of cell cycle distribution; in particular, H_2_O_2_ caused a decrease of cells in the G0/G1 phase (from 60 ± 6% to 51 ± 6%), with a concomitant increase of cells in the G2/M phase (from 9 ± 1% to 19 ± 1%) (Fig. 6a). The co-incubation with HDL completely blunted the H_2_O_2_-induced shift from the G0/G1 towards the G2/M phase (Fig. 6a). PC-3 cells also displayed an increase of cell percentage in the G2/M phase from 14 ± 4% to 18 ± 1% after exposure to H_2_O_2_ (Fig. 6b). Again, the co-incubation with HDL restored the basal cell cycle profile: when compared to H_2_O_2_-treated cells, cells in the G2/M phase were reduced (6 ± 1%) and those in the G0/G1 phase increased (from 52 ± 5% to 67 ± 7%) (Fig. 6b). HDL prevented the alterations of cell cycle distribution in H_2_O_2_-treated PNT2 cells as well (Fig. S4). HDL alone did not affect cell cycle profile of LnCaP, PC-3 and PNT2 cells (Fig. 6 and Fig. S4).

### Synthetic HDL exert anti-oxidant activity on LNCaP and PC-3 cells

**Figure 7:**
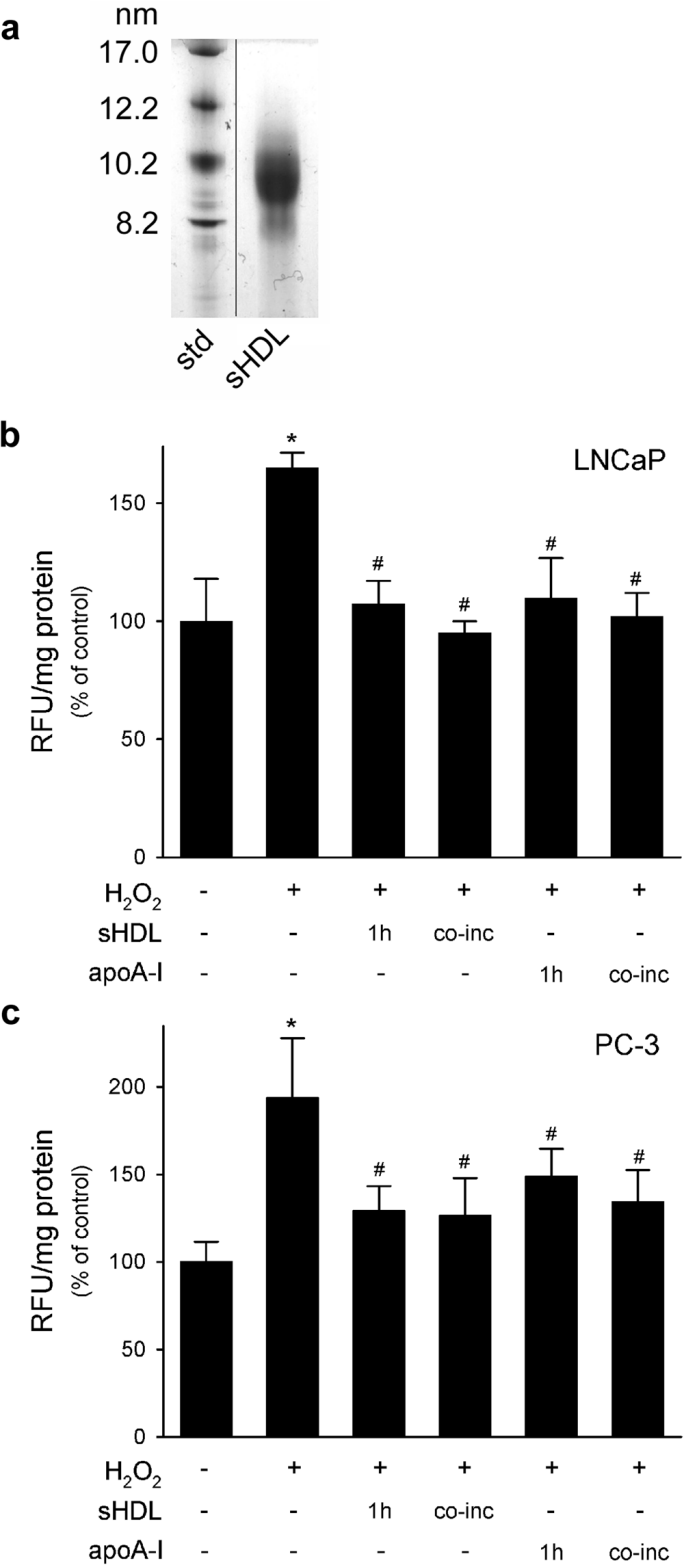
Synthetic HDL retain the anti-oxidant effect of plasma-derived HDL. Panel a, synthetic HDL particle formation and size was verified by native GGE. LNCaP (Panel b) and PC-3 cells (Panel c) were stimulated with 0.5 mM H_2_O_2_ for 1 h. sHDL or apoA-I were tested at 0.5 mg/ml as a pre-treatment of 1 h or as co-incubation with H_2_O_2_ (co-inc). ROS production was evaluated by fluorescence. Data are expressed as relative fluorescent units (RFU) normalized by the protein concentration of total cell lysate, mean ± SD, n = 4. *P < 0.05 vs control, ^#^*P* < 0.05 vs H_2_O_2_-stimulated cells.

Synthetic HDL consisted in a single population of discoidal particles with a diameter of 9.7 nm; unlipidated apoA-I was undetectable at the bottom of the gel (Fig. 7a). Based on final weight ratio, each sHDL contained 148 POPC and 2 apoA-I molecules per particle.

Synthetic HDL were able to inhibit ROS production when given in combination with H_2_O_2_ in both LnCaP (−42%) and PC-3 cells (−35%, Fig. 7). sHDL were also effective when given for 1 h and then removed prior to H_2_O_2_ stimulation; ROS production was indeed reduced by 35% in LNCaP and by 33% in PC-3 cells (Fig. 7).

Unlipidated apoA-I was also tested in both cell lines. In LNCaP, apoA-I was as effective as sHDL, since it was able to completely blunt H_2_O_2_-induced rise in ROS levels when given before or as co-incubation with the pro-oxidant stimulus (Fig. 7b). In PC-3 cells, apoA-I was as effective as sHDL when given as a co-incubation with H_2_O_2_ (−30.7%). On the contrary, when cells were pre-treated with apoA-I and then stimulated with H_2_O_2_, ROS levels were reduced by 23.2%, but they were still significantly elevated compared to baseline values (Fig. 7c).

## Discussion

We showed for the first time that HDL are able to blunt PCa cell proliferation induced by oxidative stress *in vitro*. In particular, HDL reduced ROS levels in AR-positive and AR-null cellular lines of prostate cancer, thus inhibiting ROS-induced cell entry in the G2/M phase. Interestingly, synthetic HDL made of apoA-I and phosphatidylcholine fully retained the antioxidant potential of plasma-derived HDL.

The relevance of oxidative stress to PCa development, progression and conversion to a castration-resistant phenotype has been demonstrated *in vitro* and *in vivo*^6^. ROS are generated within the cells by mitochondria and by extramitochondrial enzymatic systems as NADPH oxidases, and in tumour microenvironment by xenobiotics or inflammatory cells^9^. Interestingly, we first confirmed that PC-3 cells, which resemble the more aggressive PCa phenotype, display higher ROS levels than androgen-sensitive and non-tumorigenic ones^8^; then, we showed that short- or long-term exposure to HDL reduced basal oxidative stress in both AR-positive and AR-null cell lines. This effect could be relevant to avoid adaptation to oxidative stress-based therapeutics, as radiation^21^. Indeed, it has been described that high levels of ROS can activate the anti-oxidative machinery leading cells to be less sensitive to toxic effects of ROS generated by radiation or other similar therapies; thus, by reducing basal oxidative stress of PCa cells, HDL may facilitate the action of stress-based therapies. In addition, the antioxidant potential of HDL was tested in an extreme condition, as an acute challenge with hydrogen peroxide; we showed that HDL were able to limit H_2_O_2_-induced oxidative stress and, consequently, oxidative stress-induced proliferation in both PCa cells lines. HDL were effective not only when given in combination with the pro-oxidant stimulus but also when added before it, even for only 1 h; thus, HDL were able to rapidly modify PCa cells, making them more resistant to the following pro-oxidant stimuli. Furthermore, the modulation of the expression of proteins involved in the redox balance could be excluded in our experimental setting, since HDL were effective after only 1 h. The reduction of ROS levels is likely due to (i) HDL protein and lipid components that, acting at cell surface or within the cells, are able to directly turn off the oxidative cascade, or (ii) HDL-mediated inhibition of pro-oxidant enzymes as NADPH oxidases, whose key role in ROS generation was recently shown in PCa cells^22^. Of note, HDL were effective at concentrations well below their physiological level in plasma; indeed, 0.5 mg/ml of total HDL protein roughly corresponds to 40 mg/dl of apoA-I (since it accounts for the 80% of HDL proteins) and 10 mg/dl of HDL-cholesterol in plasma^23^.

The interaction between HDL and membrane transporters/receptors, as the ATP-binding cassette transporters A1 and G1 and the scavenger receptor type BI, is a key step in HDL-mediated modulation of cell cholesterol content, but evidence suggests its involvement in many other atheroprotective activities of HDL^24^. Thus, the expression of these transporters and receptors was analysed in the two PCa cell lines compared to non-tumorigenic cells. ABCA1 was expressed at very low levels in normal prostate epithelial cells and a further decrease was observed in both LNCaP and PC-3 cells; this reduction could be due to the marked hypermethylation at the *ABCA1* promoter site, as previously shown^25^. On the contrary, we showed that ABCG1 protein expression was markedly upregulated in tumour cell lines, especially in the AR-null one; the mechanisms and the consequences of ABCG1 upregulation on the metabolism of PCa cells deserve further investigations. The scavenger receptor BI is abundantly expressed in all cell lines at similar levels. However, neither ABCG1 nor SR-BI were involved in HDL-mediated antioxidant effect on PCa cells, as shown by silencing experiments. The role of cell cholesterol modulation on antioxidant activity of HDL was further excluded by the pre-treatment with LDL or βMCD. The lack of SR-BI involvement could be particularly relevant, since its role in tumorigenesis is debated^26^. Indeed, this receptor can facilitate a bidirectional flux of cholesterol between cells and HDL, according to the concentration gradient. Indeed, spherical and large discoidal HDL can accept cellular cholesterol through SR-BI; on the contrary, cholesteryl esters-rich HDL can become cholesterol donors via this pathway^27–29^. Thus, SR-BI could favour cholesterol uptake by cancer cells and, in addition, the interaction of HDL with SR-BI could activate the PI3K/Akt and Erk1/2 pathways promoting cell proliferation^30^. Accordingly, in prostate cancer cells the inhibition of SR-BI expression was shown to reduce cell viability^31^ and a positive correlation was found between elevated SR-BI expression and tumour grade, metastasis and poorer patient outcomes^32^. Experimental evidence suggests that AR activation may mediate oxidative stress in PCa cells^21,33,34^. To address the possible involvement of AR signalling, the non-steroidal AR antagonist bicalutamide was used; when given overnight to LNCaP cells, bicalutamide did not affect the extent of ROS generation by H_2_O_2_ or the antioxidant potential of HDL, thus excluding a role of AR activation in our experimental setting.

Overall, even if obtained only in two of the several available PCa cells lines, our results provide the rationale for investigating the relevance of HDL as a therapeutic tool against prostate cancer. In particular, HDL could have a role as adjuvant agents, able to make cancer cells more sensitive to classic cytotoxic molecules. However, the *in vivo* relevance of our findings is unknown; a prostate tumour xenograft model will be needed to address this issue and to confirm that HDL are able to affect the proliferation of both androgen-dependent and castration-resistant PCa cells. In addition, even if cell cycle analysis did not shown a change in the percentage of apoptotic cells, it is not possible to exclude that the decrease of cell number detected after treatment with HDL is due to other effects than an inhibition of cell proliferation.

HDL are not suitable for the development as drugs; they are too heterogeneous and their isolation from human plasma poses key safety concerns. Synthetic HDL made of the main protein and phospholipid components of HDL, namely apoA-I and phosphatidylcholine, were shown to retain most of plasma-derived HDL activities *in vitro* and *in vivo* and are currently in the clinical phase of development as anti-atherosclerotic agents in the context of acute coronary syndrome^18^. These synthetic particles are different from the majority of HDL circulating in plasma. First, they display a discoidal and not a spherical shape: discoidal HDL are present in plasma, they are nascent particles called preβ-HDL and usually account for the 10–15% of total HDL. Second, their protein and lipid composition is very simple and, in particular, they do not contain cholesterol, which is not considered responsible for any anti-atherosclerotic effect of HDL^17^. An advantage of sHDL use is that they are “customizable”, as their shape, size and protein/lipid composition can be modulated to achieve maximal effect. For example, if particle diameter is kept below 9.6 nm, the interaction with SR-BI could be avoided^29^. In addition, sHDL particles could also be used as carriers of therapeutically relevant molecules to be targeted to cancer cells. We showed that sHDL retained the antioxidant capacity of plasma-derived HDL, thus strengthening the idea that sHDL could be investigated as adjuvant anti-tumoral agents. Indeed, when used at the same protein concentration, their antioxidant potential was equal to that of plasma-derived HDL. However, the actual HDL activity could have been underestimated since the isolation process severely reduced the activity of antioxidant enzymes as PON and LCAT. ApoA-I was the active component of sHDL, even if protein lipidation was necessary for maximum antioxidant effect on PC-3 cells. For a possible therapeutic application, apoA-I lipidation is mandatory to avoid rapid renal excretion.

In the present paper, we have analysed HDL effects related to their anti-oxidant properties. However, HDL are able to exert a series of effects that could contribute to limit cancer cell proliferation, as their anti-inflammatory capacity or their ability to reduce cell cholesterol content^15,16^. To address these issues is beyond the scope of the present study and dedicated investigations are ongoing. Other studies evaluated the effect of HDL on different cancer cell types including PCa cells with contrasting results. In particular, two studies on PCa cells found an increase of proliferation after exposure to HDL^31,35^. However, differently from our experimental setting, proliferation assays were performed with serum-poor medium, thus it is conceivable that in such condition, HDL could represent the only source of cholesterol for almost starved cells, as suggested by Angius *et al*.^36^. On the contrary, our proliferation experiments were performed with regular growth medium including 5% or 10% FBS and no increase of proliferation (or even a slight reduction) was observed in HDL-treated cells not stimulated with hydrogen peroxide. Consistently, it has been recently shown that VLDL and LDL, but not HDL, increased viability of breast cancer cells maintained in 10% FBS^37^.

In light of the proposed anti-tumoral role for HDL, it is reasonable to query whether an association between plasma levels of HDL-C and the incidence or the progression of PCa exists. Several studies examined the possible correlation between HDL levels (measured as plasma levels of HDL-C or of apoA-I, the main protein component of HDL) and cancer risk. The issue is still debated since both positive and negative associations were found, likely due to the presence of confounding factors as concomitant pathologies and/or therapies; in addition, patients with cancer usually display a disease-related reduction of plasma HDL-C. However, there are several indications from observational and interventional studies that support the anti-tumoral role of HDL (recently reviewed in^38^). Plasma apoA-I and HDL-C were inversely related to the risk of different types of cancers as ovarian, breast, colon, lung and prostate, with a recent meta-analysis showing a 36% risk reduction for every 10 mg/dl increase of plasma HDL-C levels^39^. Consistently, plasma apoA-I levels were included in a FDA-approved test for early stage ovarian cancer, together with transthyretin, transferrin, β2-microglobulin and CA125^40^. To support a causative association, human apoA-I transgenic mice displayed reduced lung, melanoma or ovarian tumour development compared to control animals, an effect also observed in mice infused with human apoA-I^41,42^. As learned from studies on their atheroprotective activities, HDL are heterogeneous and different subclasses can display different activities^43^. In addition, several physiologic and pathologic conditions can modify HDL composition and function. Interestingly, HDL from patients with type 2 diabetes and oxidized HDL, but not control HDL, induced proliferation of breast cancer cells^44,45^. Further investigations are required to assess whether cancer itself or anti-tumoral therapies may affect HDL functions other than their plasma levels.

## Materials and Methods

### Lipoproteins

LDL (d = 1.020–1.063 g/ml) and HDL (d = 1.063–1.21 g/ml) were purified by sequential ultracentrifugation from the plasma of healthy volunteers^46^. Total and unesterified cholesterol (TC and UC), triglycerides, phospholipids, apolipoprotein A-I and A-II levels in isolated HDL were determined with standard enzymatic or turbidimetric techniques by using a Roche c311 analyzer (Roche Diagnostics, Germany). The cholesteryl ester mass was calculated as (TC − UC) × 1.68. HDL composition was expressed as percentage of total HDL mass^47^. LCAT concentration was measured by a specific competitive ELISA developed within our laboratory and LCAT activity was evaluated as the ability to esterify cholesterol incorporated into an exogenous standardized substrate, as described^48^. PON activity was measured as arylesterase activity using phenyl acetate (Sigma-Aldrich, Germany) as substrate^49^. Apolipoprotein A-I was purified from human blood plasma by gel filtration chromatography as previously described^50^.

The study conformed to the guidelines set out in the Declaration of Helsinki and pertinent ethical regulations. The protocol was approved by the ethical committee of Niguarda Hospital (n. 451-092014) and all volunteers gave written informed consent for participation in the study. sHDL containing synthetic palmitoyl-oleoyl-phosphatidylcholine (POPC, Sigma-Aldrich Chemie GmbH, Germany) and apolipoprotein A-I were prepared by the cholate dialysis technique, with a starting POPC:protein weight ratio of 2.17:1^51^. The size of sHDL was estimated by nondenaturing gradient gel electrophoresis, using the Pharmacia Phast System^52^. Protein concentration was evaluated by the method of Lowry. HDL and sHDL preparations were dialyzed against sterilized saline immediately before use and their concentrations are expressed as protein content.

### Cell lines

Experiments were performed using two different human epithelial cell models of prostatic tumour, namely LNCaP and PC-3, and the human normal prostate epithelial cell line PNT2. LNCaP are AR positive and sensitive and were shown to be tumorigenic in nude mice with a low metastatic potential^53^. On the contrary, PC-3 are AR-null and display an androgen-independent growth; they were shown to be tumorigenic with a high angiogenic and metastatic potential in nude mice^53^. SV40-immortalized PNT2 are non-tumorigenic human prostatic epithelial cells^54^. Tumour cell lines were purchased from ATCC (VA, USA) and PNT2 were purchased from Sigma-Aldrich (Germany); cells were maintained in RPMI 1640 supplemented with 5% (PC-3) or 10% (LNCaP and PNT2) fetal bovine serum, 1% penicillin/streptomycin and 2 mM L-glutamine at 37 °C in a humidified atmosphere with 5% CO_2_. Confluent cells were harvested with 0.05% trypsin/0.02% EDTA and seeded in Petri dishes or multiwell plates, as indicated below. Cell media, supplements and sterile disposables were purchased from EuroClone (Italy).

### ROS assay

PNT2, LNCaP and PC-3 cells seeded in 24-well black plates were incubated with HDL, sHDL or apoA-I at 0.5 mg/ml up to 16 h in RPMI 1640 (supplemented with 1% bovine serum albumin for LNCaP). Cells were then washed with PBS and loaded with 3 μM 2′,7′-dichlorofluorescein (Life Technologies, USA) for 30 minutes. After RPMI 1640 replenishment, cells were stimulated or not with 0.5 mM H_2_O_2_ for 1 h and ROS production was measured by fluorescence on a Synergy H1 multi-mode reader equipped with the Gen5 software (BioTek, USA). To evaluate the role of AR signalling in ROS generation and HDL antioxidant effect, in separate experiments LNCaP were maintained in RPMI 1640 without phenol red and incubated overnight with the AR antagonist bicalutamide (100 nM, Sigma-Aldrich). To evaluate the role of cell cholesterol content on HDL antioxidant effect, cells were pre-treated overnight with LDL (50 μg/ml) or for 1 h with 2.5 mM beta-methylcyclodextrin (βMCD, Sigma Aldrich, Germany).

For each sample, fluorescence was normalized by the protein concentration of the total cell lysate, measured by the micro-bicinchoninic acid assay (Thermo Scientific, USA).

A standard HDL preparation from pooled plasma was tested in each experiment for inter-assay data correction.

### Cell cholesterol content

Cells were washed with PBS and lysed overnight in 1% sodium cholate and 10 U/ml DNase (Sigma Aldrich, Germany). Cholesterol was measured by fluorescence using the Amplex Red Cholesterol Assay Kit (Sigma Aldrich, Germany), according to the manufacturer instructions^55^. For each sample, cholesterol concentration was normalized by the protein concentration of the total cell lysate, measured by the micro-bicinchoninic acid assay.

### Western Blotting

PNT-2, LNCaP and PC-3 cells were lysed in 20 mmol/L Tris buffer containing 4% SDS, 20% glycerol, 1 mmol/L EDTA, 1 mmol/L sodium orthovanadate, 1 mmol/L NaF, 1 μg/mL leupeptin, 1 mmol/L benzamidine, 10 μg/mL soy trypsin inhibitor, 1 mmol/L PMSF, and 0.5 mmol/L DTT. Cell debris was removed by centrifugation and protein concentration was determined by the micro-bicinchoninic acid assay. Proteins were separated by 10% SDS-PAGE and transferred on a nitrocellulose membrane. Membranes were developed against ABCA1, ABCG1 and SR-BI (Novus Biologicals, USA), stripped and reprobed with an antibody against β-actin (Sigma Aldrich, Germany). Further details are provided in Table S2. Bands were visualized by ECL (GE Healthcare Biosciences, Sweden) and band densities were evaluated with a GS-690 Imaging Densitometer equipped with the Multi-Analyst software (Bio-Rad Laboratories).

### Silencing of SR-BI and ABCG1

PC-3 cells were seeded in 24-well black plates and transfected with 100 pmol of siRNA against either SR-BI, ABCG1 or noncoding (scrambled) siRNA for 48 h using the OptiMEM/Lipofectamine 2000 system (Life Technologies, UK), according to the manufacturer’s protocol. siGenome ON-TARGETplus SMARTpool were used (Dharmacon, ThermoScientific, USA). Cells were then washed with PBS and ROS assay was performed as described above. Silencing efficiency was evaluated by western blotting for ABCG1 or SR-BI and β-actin, as described above.

### Cell proliferation

LNCaP and PC-3 cells were seeded (300.000 and 100.000 cells/well, respectively) in 6-well culture plates in RPMI containing 5–10% FBS (see above). After 48 h cells were moved to RPMI 1640 without phenol red containing 5 or 10% charcoal-stripped FBS and allowed to proliferate for additional 48 h. Medium was then refreshed and cells were treated for 72 h with saline, 5 µM H_2_O_2_^56^, HDL (0.5 mg/mL) or with a combination of both. To evaluate the impact of treatments on cell proliferation, cells were harvested and counted by Trypan blue exclusion in a Burker chamber. Cell growth was further analysed by the MTT assay (Promega, USA), according to manufacturer instructions. After 4 h at 37 °C, absorbance at 570 nm was measured with the Synergy H1 multi-mode reader equipped with the Gen5 software (BioTek, USA).

### Cell cycle analysis

Flow cytometry was used to analyse cell cycle distribution. After trypsinization LNCaP and PC3 were re-suspended in 70% cold EtOH (700 μL) and fixed on ice for 15 min. Cells were then centrifuged, the pellet was washed with cold PBS Ca^2+^/Mg^2+^ and re-suspended in a solution containing 40 μg/mL of RNAse and propidium iodide (5 μM). Samples were kept in the dark for 15 min, and the fluorescence of individual nuclei was measured. Data acquisition was performed in a NovoCyte flow cytometer (ACEA Biosciences, USA) using NovoExpress 1.0.2 software. A minimum of 10,000 events/sample were counted. The number of cells in G0/G1, S, and G2/M phases was expressed as percentage of total events.

### Statistical analysis

Results are reported as mean and standard deviation (SD). Group differences were evaluated by non parametric Kruskal-Wallis and Mann-Whitney tests. The α level of significance was set at 0.05. All experiments were performed at least three times using different preparations of HDL or sHDL. Statistical analysis was performed using SPSS version 24.0 software (SPSS Inc., Chicago, USA).

## Electronic supplementary material

Supplementary results
