# Supplementary material for: High Density Lipoproteins Inhibit Oxidative Stress-Induced Prostate Cancer Cell Proliferation
Source: Sci Rep. 2018 Feb 2;8:2236. doi: 10.1038/s41598-018-19568-8 (PMC5797231; doi:10.1038/s41598-018-19568-8)

HIGH DENSITY LIPOPROTEINS INHIBIT OXIDATIVE STRESS-INDUCED PROSTATE CANCER CELL PROLIFERATION

Massimiliano Ruscica[1*], Margherita Botta[1*], Nicola Ferri[2], Eleonora Giorgio[3], Chiara Macchi[1], Guido Franceschini[3], Paolo Magni[1], Laura Calabresi[3] and Monica Gomaraschi[3]

[1]*Dipartimento di Scienze Farmacologiche e Biomolecolari, Università degli Studi di Milano, Milano, Italy*

[2]*Dipartimento di Scienze del Farmaco, Università degli Studi di Padova, Padova, Italy*

[3]*Centro Enrica Grossi Paoletti, Dipartimento di Scienze Farmacologiche e Biomolecolari, Università degli Studi di Milano, Milano, Italy*

SUPPLEMENTARY INFORMATION FILE

TABLE S1. Composition of isolated HDL

Composition	% of total HDL mass
Proteins	42.8±2.9
Phospholipids	23.4±3.0
Cholesteryl esters	26.7±2.0
Unesterified cholesterol	4.2±0.9
Triglycerides	2.9±0.7
Proteins	% of total HDL protein
Apolipoprotein A-I	78.1±3.2
Apolipoprotein A-II	18.4±6.3

Data are expressed as mean±SD, n=6.

TABLE S2. ANTIBODIES

Target	Company	Code	Type	Host	Dilution
Human ABCA1	Novus biologicals	NB400-105	Polyclonal	Rabbit	1:1000
Human ABCG1	Novus biologicals	NB400-132	Polyclonal	Rabbit	1:1000
Human SR-BI	Novus biologicals	NB400-104	Polyclonal	Rabbit	1:1000
Actin	Sigma-Aldrich	A5060	Polyclonal	Rabbit	1:2000
Rabbit immunoglobulins	DakoCytomation	P0448	Polyclonal HRP-conjugated	Goat	1:2000

Figure S1

Oxidative stress evaluation. Basal oxidative stress in PNT2, LNCaP and PC-3 cells. Data are expressed as relative fluorescent units (RFU) normalized by the protein concentration of total cell lysate, mean±SD, n=4.*P<0.05 vs PNT2, $^{\#}P$<0.05 vs LNCaP.

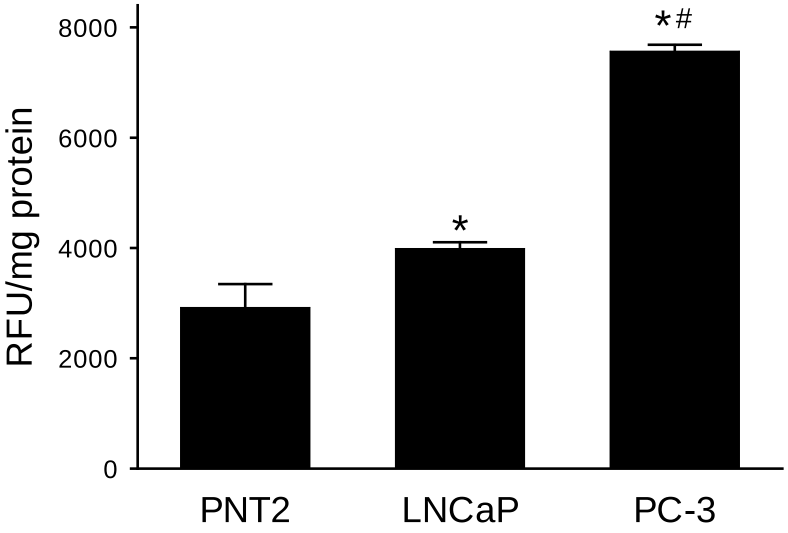

Figure S2

Concentration-dependent effect of HDL on ROS production in LNCaP cell line. LNCaP were co-incubated with 0.5 mM H_2O_2 and increasing concentrations of HDL for 1h. ROS production was evaluated by fluorescence. Data are expressed as relative fluorescent units (RFU) normalized by the protein concentration of total cell lysate, mean±SD, n=5. *P<0.05 vs control, #P<0.05 vs H_2O_2-stimulated cells.

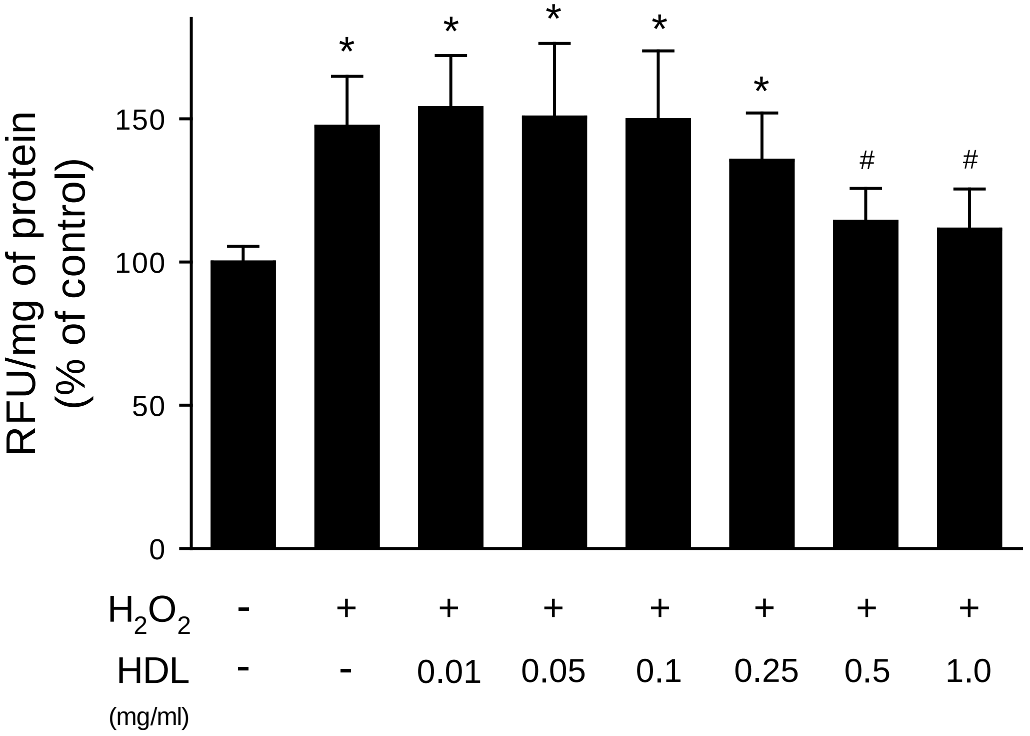

Figure S3

Antioxidant effect of HDL is independent from the modulation of cell cholesterol content. LNCaP (upper panel) and PC-3 (lower panel) were pre-treated with 50 μg/ml of LDL overnight or with 2.5 mM βMCD for 1h. Cells were then stimulated with 0.5 mM H_2O_2 in the presence or absence of 0.5 mg/ml of HDL, as indicated. ROS production was evaluated by fluorescence. Data are expressed as relative fluorescent units (RFU) normalized by the protein concentration of total cell lysate, mean±SD, n=4. *P<0.05 vs unstimulated cells with the same pre-treatment, #P<0.05 vs H_2O_2-stimulated cells with the same pre-treatment.

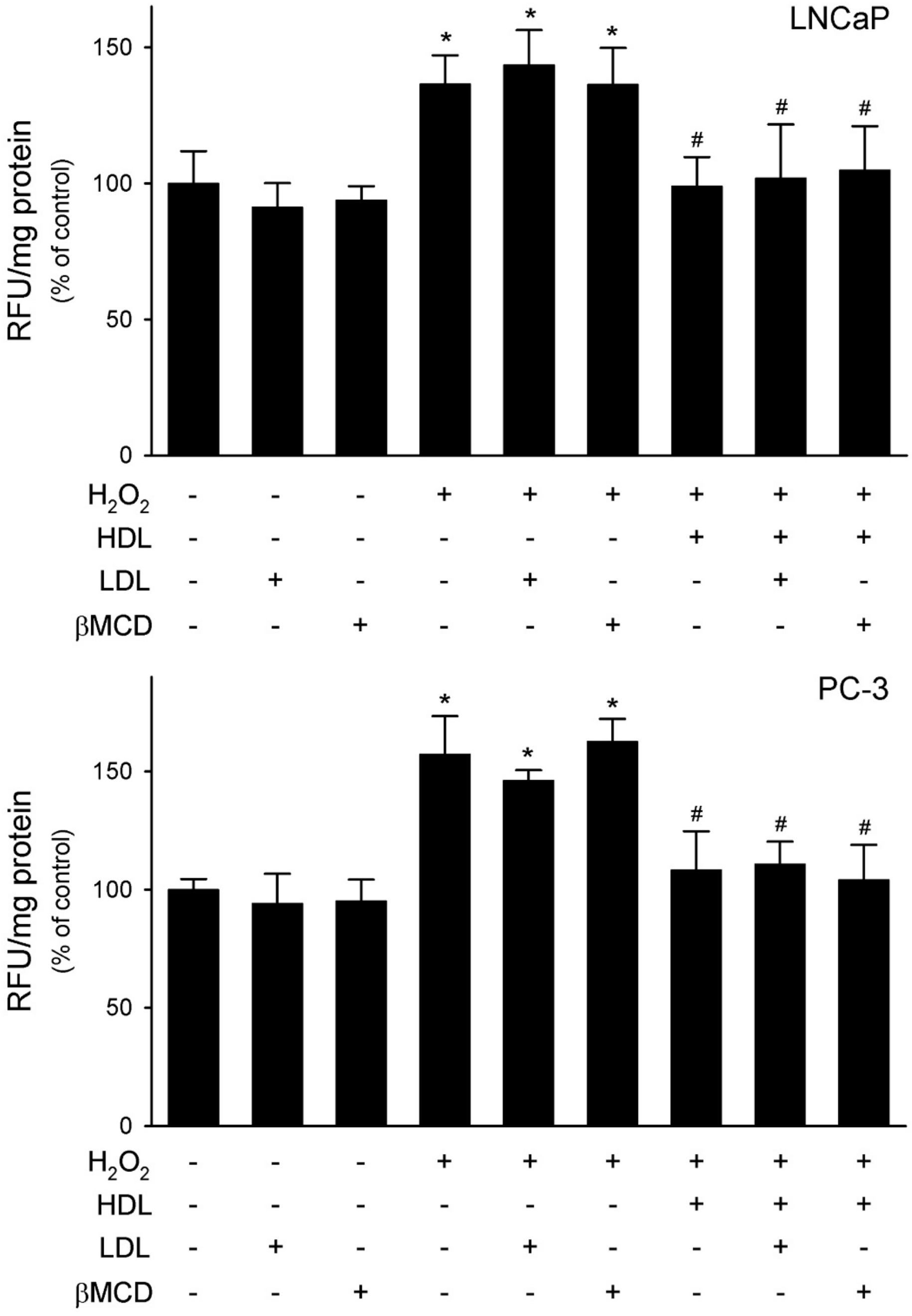

Figure S4

HDL reduce ROS production and modulate cell cycle distribution in PNT2 cell line. Panel A, PNT2 were pre-treated overnight (o/n) or for 1h with HDL 0.5 mg/ml and then stimulated or not with 0.5 mM H_2O_2 for 1h. The effect of the co-incubation of HDL with H_2O_2 (co-inc) was also tested. ROS production was evaluated by fluorescence. Data are expressed as relative fluorescent units (RFU) normalized by the protein concentration of total cell lysate, mean±SD, n=4. **P*<0.05 vs control, $^{\#}$*P*<0.05 vs H_2O_2-stimulated cells. Panel B, PNT2 were incubated for 72h with H_2O_2 5μM, HDL 0.5 mg/ml or with a combination of both. Cells were then harvested, stained with propidium iodide and subjected to FACS analysis. Cumulative results of the percentage distribution along cell cycle (n=3) are shown.

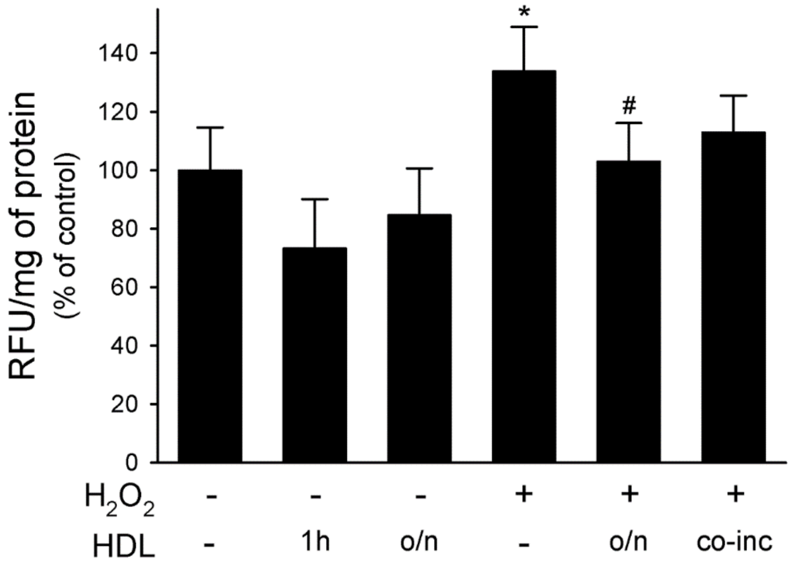

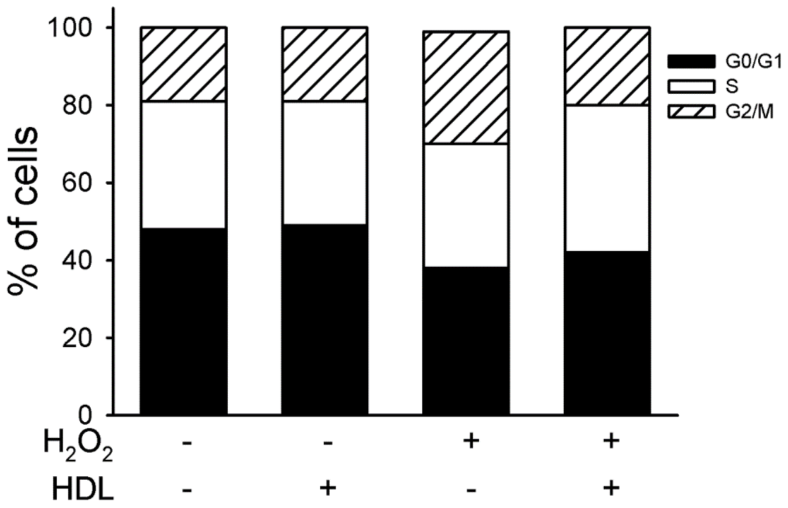

Figure S5.

Original blots of figures 3, 4 and 7

Original images for ABCA1, ABCG1, SR-BI and actin expression in PNT-2, LNCaP and PC-3. The manipulated versions are reported in figure 3 of the manuscript.

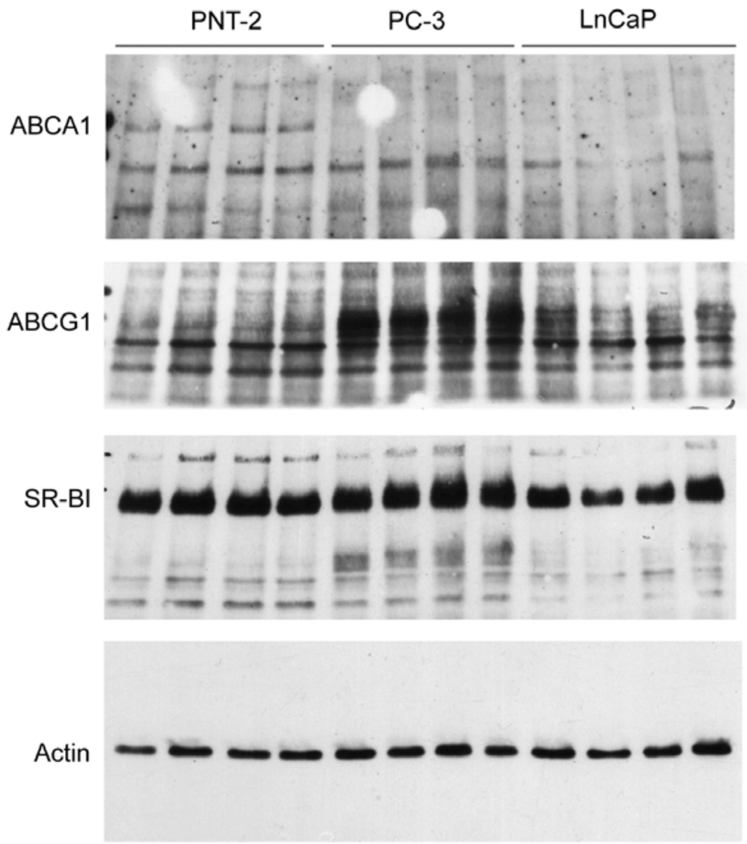

Original images for SR-BI, ABCG1 and actin blots of scrambled, SCARB1- or ABCG1-treated PC-3. The manipulated versions are reported in figure 4 of the manuscript.

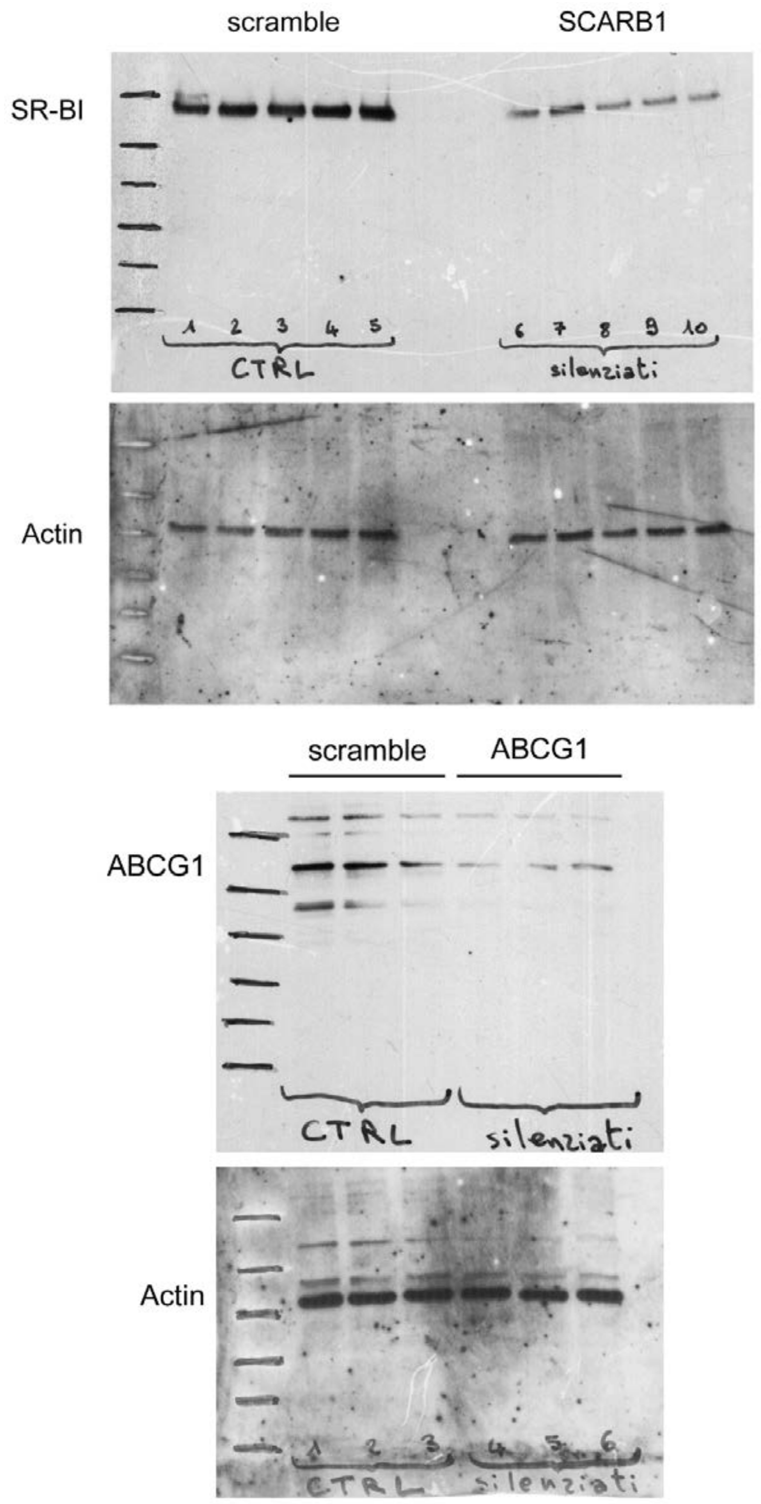

Original images for GGE of sHDL. Products 1 and 2 were verified on the same gel, but not used in the present work. The manipulated version is reported in figure 7 of the manuscript.

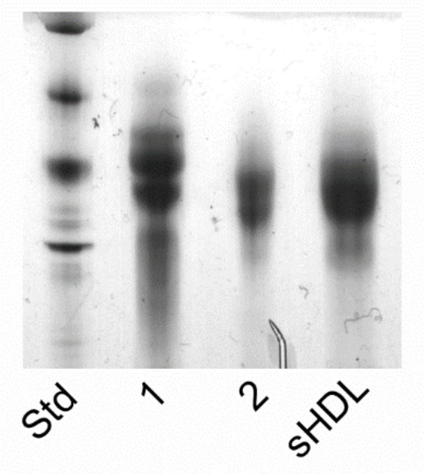

Supplement: Supplementary file 1 — Supplementary results [file 41598_2018_19568_MOESM1_ESM.pdf]
